# Hypoxia Induces an Immunodominant Target of Tuberculosis Specific T Cells Absent from Common BCG Vaccines

**DOI:** 10.1371/journal.ppat.1001237

**Published:** 2010-12-23

**Authors:** Hannah Priyadarshini Gideon, Katalin Andrea Wilkinson, Tige R. Rustad, Tolu Oni, Heinner Guio, Robert Andrew Kozak, David R. Sherman, Graeme Meintjes, Marcel A. Behr, Hans Martin Vordermeier, Douglas Brownlee Young, Robert John Wilkinson

**Affiliations:** 1 Institute of Infectious Diseases and Molecular Medicine, University of Cape Town, Observatory, South Africa; 2 MRC National Institute for Medical Research, Mill Hill, London, United Kingdom; 3 Seattle Biomedical Research Institute, Seattle, Washington, United States of America; 4 Division of Medicine and Center for Molecular Microbiology and Infection, Imperial College London, London, United Kingdom; 5 McGill University Health Centre, Montreal, Canada; 6 Veterinary Laboratories Agency, Weybridge, United Kingdom; University of New Mexico, United States of America

## Abstract

*M. tuberculosis* (MTB) species-specific antigenic determinants of the human T cell response are important for immunodiagnosis and vaccination. As hypoxia is a stimulus in chronic tuberculosis infection, we analyzed transcriptional profiles of MTB subject to 168 hours of hypoxia to test the hypothesis that upregulation by hypoxia might result in gene products being recognized as antigens. We identified upregulation of two region of difference (RD) 11 (Rv2658C and Rv2659c), and one RD2 (Rv1986) absent from commonly used BCG strains. In MTB infected persons, the IL-2 ELISpot response to Rv1986 peptides was several times greater than the corresponding IFN-γ response to the reference immunodominant ESAT-6 or CFP-10 antigens. The IL-2 response was confined to two epitopic regions containing residues 61–80 and 161–180. The biggest population of IL-2 secreting T cells was single cytokine positive central memory T cells. The IL-2 response to live MTB bacilli lacking Rv1986 was significantly lower than the response to wild type or mutant complemented with Rv1986. In addition, the IL-2 response to Rv1986 was significantly lower in HIV-TB co-infected persons than in HIV uninfected persons, and significantly increased during antiretroviral therapy. These findings demonstrate that Rv1986 is an immunodominant target of memory T cells and is therefore of relevance when considering the partial efficacy of currently used BCG vaccines and provide evidence for a clinical trial comparing BCG strains.

## Author Summary

*Mycobacterium tuberculosis* (the cause of tuberculosis) can persist for many years in humans without causing disease but has the potential to reactivate. One of the conditions the bacterium must survive in these circumstances is hypoxia. In order to do so, the bacterium uses a characteristic set of genes that help alter its metabolism. It follows that the products of such genes may encode protein antigens that can be recognized by the immune response. We therefore analyzed gene response patterns of tuberculosis subject to prolonged hypoxia as a guide to the discovery of new antigens that might be useful as vaccines or diagnostic agents. Amongst the genes most strongly increased by low oxygen levels, one was identified (known as Rv1986) that is missing from most strains of the tuberculosis vaccine *Mycobacterium bovis* BCG. When we analyzed human immune responses to this protein in tuberculosis infected people our experiments showed it was particularly well recognized by cells that produce a chemical messenger (cytokine) called interleukin-2. Interleukin-2 is important for long-term immunological memory. The BCG vaccine is only partially effective and our experiments therefore suggest one of the reasons could be that an important immunological target is missing from many strains. Further evaluation of BCG strains in which Rv1986 is present or absent is therefore warranted in the hope that this might improve the efficacy of existing or new tuberculosis vaccines.

## Introduction

*Mycobacterium tuberculosis* remains a formidable health problem as it is estimated to infect one-third of the world's population and causes around 1.5 million deaths per year [1]. Control is largely based around the partially effective vaccine *Mycobacterium bovis* Bacille Calmette Guérin (BCG) and on the early detection and treatment of infected persons with active or latent disease [2].

Study of the antigens of *M. tuberculosis* is therefore a priority both to improve vaccination via the selection of protective antigens, and to define immunodiagnostic candidates that enhance the specificity and sensitivity of the widely used purified protein derivative (PPD) based tuberculin skin test (TST). A significant landmark in both respects was the discovery that a *M. tuberculosis* genomic region designated region of difference (RD) 1 is deleted from all strains of BCG and thereby partially accounts for the avirulence of the vaccine [3], [4]. RD1 encodes a pair of co-regulated secreted proteins (ESAT-6 and CFP-10) that are highly immunogenic [5], [6]: restoration of these genes into BCG improves its vaccine efficacy [7]. Assays of the T cell interferon (IFN)-γ secretion in response to the combination of ESAT-6 and CFP-10 (interferon-γ release assays, IGRA) have been developed that have operational advantages and improve the specificity and possibly sensitivity of tuberculosis immunodiagnosis [8].

The availability of the complete sequence of *M. tuberculosis* also permitted further genomic characterization of various BCG strains [9], [10], [11]. It became apparent that, against a background of accumulating single nucleotide polymorphisms, BCG underwent sequential genomic deletions that thereby characterize various strains. The strains most commonly in use such as BCG Glaxo, Danish and Pasteur have most deletions. This led to the proposal that one of the reasons behind the partial vaccine efficacy of BCG was that it had become too attenuated to successfully mimic natural MTB infection [12]. Some empirical evidence in humans favoring this hypothesis is provided by the finding that BCG Japan induced greater cytotoxic and T helper 1 responses in infants than Danish BCG [13]. The largest difference between BCG Japan and BCG Danish is the presence of RD2 in the former but not the latter.

The discovery of immunodominant antigens in *M. tuberculosis* has hitherto largely been based on dominance in antibody responses that are neither the basis of protection against tuberculosis nor of IGRA. A more rational approach might be to relate what is highly expressed by bacilli *in vivo* or *in vitro* (and thereby potentially available as an antigen) as recently investigated in bovines [14]. In humans there has been investigation of proteins encoded by genes of the *dos*R regulon that is induced in axenic culture by hypoxia and by nitric oxide [15], stresses that are considered relevant to bacilli in nature [16], [17], [18]. Analysis of selected *dos*R regulated proteins confirmed the immunodominance of α-crystallin 1 (Acr1) encoded by Rv2031 [19], [20], [21], as well as potentially infection stage specific antigens [19], [22], [23], [24].

It has been shown *in vitro* that the up regulation of *dos*R regulated genes represents an early somewhat transient response to hypoxia: upregulation of a larger group of genes characterizes the hypoxic time course occurs in cultures subject to 4–7 days (as opposed to 2 hours) hypoxia [25], of which 230 are defined as the enduring hypoxic response (EHR). We hypothesized that these EHR and other hypoxia-induced genes would be worthy of consideration as antigens, especially those that were species-specific. We therefore undertook a study of the immunogenicity of *M. tuberculosis* specific genes induced by hypoxia and other well-characterized antigens (ESAT-6, CFP-10, Acr1) in humans with active and latent tuberculosis.

## Results

### Antigen selection

Cross-reference of genes with the greatest fold induction in hypoxic culture over 7 days [25] with sequence databases revealed two species-specific (Rv2658c and Rv2659c, both RD11 encoded) and one partially species-specific gene (Rv1986, RD-2 encoded). The fold induction and *sig*A normalized transcript intensity over a time course of 168 hrs hypoxia for these genes (and of Acr1, CFP-10 and ESAT-6) are shown in Table 1. Interestingly whilst the fold induction for the RD1 encoded genes fell, the normalized intensity remained at a similar absolute level to that of both EHR and the *dos*R regulated Acr1 gene.

**Table 1:** *M. tuberculosis* transcript levels in bacilli exposed to 7 days hypoxia *in vitro.*

		Log base2 Hypoxia/Log (a)				Median transcript intensity normalized to SigA (b)			
Rv#	Gene name	4 hrs	1 day	4 days	7 days	4 hrs	1 day	4 days	7 days
Rv3875	ESAT-6	−0.34	−2.17	−1.88	−1.40	5.97	4.22	3.49	4.33
Rv3874	CFP-10	0.14	−1.20	−0.56	−1.03	7.42	2.3	1.97	4.04
Rv2659c	Rv2659c	1.49	3.56	3.43	3.29	0.14	1.66	1.47	0.72
Rv2658c	Rv2658c	2.15	4.68	4.93	4.29	0.18	1.69	1.25	0.66
Rv2031c	Acr1	5.34	4.02	4.95	4.22	4.09	1.84	0.63	1.40
Rv1986	Rv1986	1.10	3.77	3.60	3.06	0.21	2.38	2.10	1.35

(a) Shows the fold induction over hypoxic time course with reference to phase of aerobic cultures.

(b) Shows the median transcript intensity normalized to *Sig*A over hypoxic time course.

### Interferon-γ ELISpot analysis of active and latent tuberculosis

**Figure 1:**
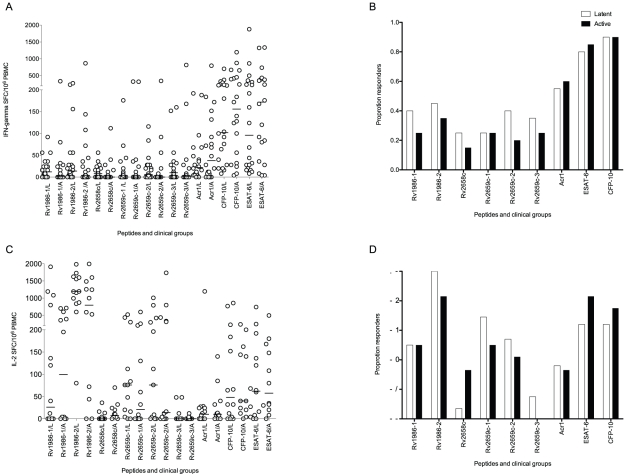
Interferon-γ and IL-2 ELISpot response to peptide pools. Panel A shows the spot forming cells (SFC) in patients with latent (L) and active (A) tuberculosis. The strongest response in latent disease was to CFP-10. With the exception of ESAT-6 all other responses were significantly lower (p≤0.007). The strongest response in active disease was to ESAT-6. With the exception of CFP-10 all other responses were significantly lower (p≤0.002). Peptide pool Rv2659c-2 was preferentially recognized by latently infected persons (p = 0.03). Panel B shows the proportion of responders (defined by a response of ≥20 SFC/10^6^ PBMC above background). No pool was preferentially recognized by either clinical group. The most frequent response in the combined group (latent plus active) was to CFP-10: with the exception of ESAT-6 the proportion subjects responding to the other antigens was lower in every case (p≤0.002). Panel C shows IL-2 spot forming cells (SFC). The strongest response in both active and latent disease was to Rv1986-2. All other responses in both latent (p≤0.0007) and active infection (p≤0.02) were significantly lower. Peptide pool Rv2658c was preferentially recognized by actively infected persons (p = 0.042). Panel D shows the proportion of responders. No pool was preferentially recognized by either clinical group. The most frequent response in the combined group (latent plus active) was to Rv1986-2: with the exception of ESAT-6 and CFP-10 the proportion subjects responding to the other antigens was lower in every case (p≤0.009). Bars show the median response.

Interferon-γ (IFN-γ) ELISpot was performed using PBMC from 40 persons with active (20) or latent (20) tuberculosis, and IL-2 ELISpot on 13 and 14 persons in each category. Immunodominance was assessed both quantitatively (median SFC/10^6^ PBMC) and by frequency of response (>20 SFC/10^6^ PBMC). CFP-10 and ESAT-6 were co-dominant for the IFN-γ response by both methods (Figure 1A and B). The largest SFC response in latent disease was to CFP-10 (102 SFC/10^6^ PBMC, IQR 38-444). With the exception of ESAT-6 all other responses were significantly lower (p≤0.007). The largest response in active disease was to ESAT-6 (172 SFC/10^6^ PBMC, IQR 47–423). With the exception of CFP-10 all other responses were significantly lower (p≤0.002). Although peptide pool Rv2659c-2 was preferentially recognized by latently infected persons (6 SFC/10^6^ PBMC, IQR 1–28 versus 0 SFC/10^6^ PBMC, IQR 0–7, p = 0.028) these responses were very modest. When analyzed by proportions, no pool was preferentially recognized by either clinical group (Figure 1B). The most frequent response in the combined group (latent plus active) was to CFP-10 (36/40, 90%): with the exception of ESAT-6 the proportion of persons responding to the other antigens was lower in every case (p≤0.002).

### IL-2 ELISpot analysis of active and latent tuberculosis

Patients with active TB preferentially recognized pooled peptides from Rv2658c (7 IL-2 SFC/10^6^ PBMC, IQR 1–23 versus 0 SFC/10^6^ PBMC, IQR 0–6 p = 0.042). However when analyzed by proportion, no pool was preferentially recognized by either clinical group. There was however a striking IL-2 response in both active and latent disease to Rv1986 pool 2 (795 SFC/10^6^ PBMC, IQR 51–1428 in active infection; 1194 SFC/10^6^ PBMC, IQR 862–1650 in latent infection, Figure 1C and D). All other antigen specific IL-2 responses were significantly lower in both latent (p≤0.0007) and active infection (p≤0.02). The most frequent response in the combined group (latent plus active) was also to Rv1986-2 (24/26, 92%): with the exception of ESAT-6 and CFP-10, the proportion of subjects responding to the other antigens was lower in every case (p≤0.009).

### Peptide mapping of the IL-2 response to Rv1986

**Figure 2:**
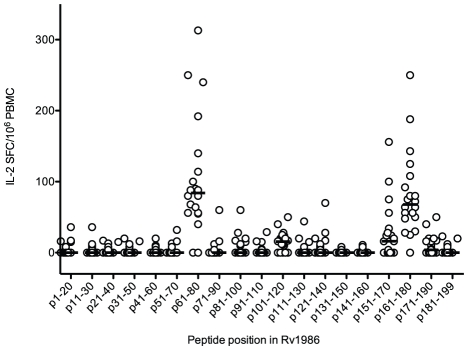
Peptide map of IL-2 response to Rv1986. PBMC from 20 subjects with latent tuberculosis were stimulated with individual peptides of Rv1986 in an IL-2 ELISpot assay. p61–80 and p161–180 were the dominant determinants of the response. Horizontal lines indicate median responses.

Epitope mapping of the individual peptide determinants of the IFN-γ response to ESAT-6 and CFP-10 have shown several regions in each molecule that can restimulate T cells with no single peptide giving rise to a response in >50% subjects [26], [27] and similar findings are reported for other antigens of *M. tuberculosis* [22]. We were therefore interested to determine whether a similarly ‘degenerate’ pattern of multiple IL-2 inducing epitopes occurred in Rv1986. PBMC from 20 persons with latent tuberculosis were assayed in the presence of 10 µg/ml of each peptide or no stimulus. A highly focused pattern of dominance was observed with peptides p61-80 (84 SFC/10^6^ PBMC, IQR 56–134) and p161–180 (68 SFC/10^6^ PBMC, IQR 49–104) being clearly the best recognized (Figure 2). 90% of subjects had a response >20 SFC/10^6^ PBMC to p61–80 and 95% to p161–180 with no other peptide being recognized by >45% subjects. There were less frequent and lower magnitude responses to p151–170 perhaps suggesting the epitope core for some MHC Class II molecules may include residues common to both peptides (161–170).

### Phenotype of CD4+ T cells responding to Rv1986

**Figure 3:**
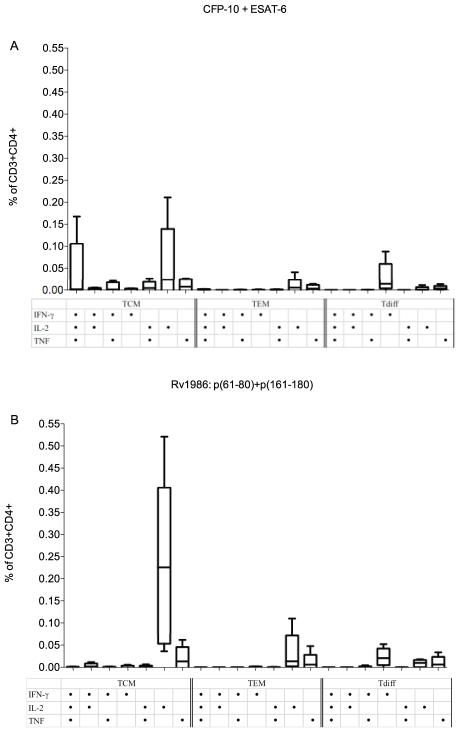
Phenotype of CD4+ T cells responding to Rv1986. PBMC from 5 donors were stimulated either with CFP-10+ESAT-6 (A) or with Rv1986 p(61–80)+p(161–180) (B), and the phenotype of T cells producing single and multiple cytokines were analyzed using surface and intracellular cytokine staining by FACS. T cell phenotype was defined by the surface markers CD45RA and CD27: Central memory cells (TCM) are positive for CD27 and negative for CD45RA; effector memory (TEM) are negative for both CD27 and CD45RA and Terminally differentiated T cells (Tdiff) are negative for CD27 and positive for CD45RA. The results are expressed as the percentage of CD3+ CD4+ T cells. The strongest response to Rv1986 p61–80+p161–180 stimuli was the IL-2 producing central memory cells, median 0.22% (Panel B); as opposed to a median of 0.02% for CFP-10 plus ESAT-6 (p = 0.055) (Panel A). While CFP-10 and ESAT-6 induced multiple cytokine-producing TCM, Rv1986 induced predominantly single cytokine producing TCM, TEM and Tdiff cells.

Analysis of the T cells responsible for type 1 cytokine responses is critical to understand protective immunity against TB [28]. In PBMC from 5 persons with latent tuberculosis, we therefore determined the phenotype of CD4+T cells responsible for type 1 cytokine (IFN-γ, IL-2 and TNF) production when stimulated with the peptides of Rv1986 (61–80 and 161–180) or the combination of peptides from CFP-10 and ESAT-6 as a comparison. T cell phenotypes were defined based on the surface markers CD45RA and CD27: Central memory cells (TCM) are positive for CD27 and negative for CD45RA; effector memory (TEM) are negative for both CD27 and CD45RA and Terminally differentiated T cells (Tdiff) are negative for CD27 and positive for CD45RA. Single cytokine positive cells predominated overall (Figure 3). Most IL-2 derived from TCM irrespective of stimulus. The two Rv1986 peptides restimulated nearly ten times the percentage of IL-2 producing TCM cells than CFP-10 and ESAT-6 (median: 0.226% CD3^+^CD4^+^ TCM vs 0.024% CD3^+^CD4^+^TCM, p = 0.055, Figure 3 panel A and B).

### Rv1986 induces a distinct pattern of cytokine secretion in addition to IL-2

**Figure 4:**
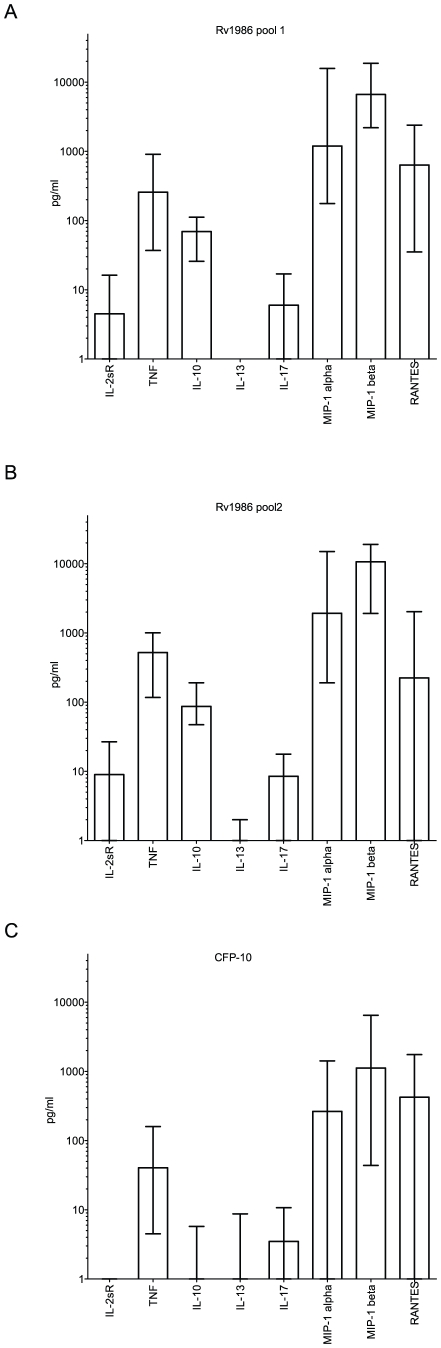
Luminex multiplex cytokine analysis for Rv1986 and CFP-10 peptide pools. Panels A, B and C show the production of different cytokines and chemokines in 39 persons with latent or active tuberculosis after 16 hrs stimulation of PBMC with peptides of Rv1986 (pools 1 and 2) and CFP-10 in cell culture supernatants respectively. The results are represented as pg/ml; bars indicate median response with IQR. The cytokine responses to Rv1986 (1 and 2) were significantly higher than that of CFP-10 for IL2sR (p<0.0001), TNF (p<0.0001), IL-10 (p<0.0001), IL-13 (p≤0.01), IL-17(p = 0.01), MIP-1alpha (p≤0.005) and MIP-1beta (p<0.004), but not RANTES (p>0.5).

We further investigated the ability of Rv1986 to induce the secretion of other cytokines when compared to CFP-10. We used 16-hour cell culture supernatants from 39 persons with either active (19) or latent (20) tuberculosis. Multiple cytokine secretion was assessed both quantitatively (pg/ml, after background correction) and by frequency of response (>2 fold above background). Similar levels of cytokine responses were observed in both analyses in persons with active and latent tuberculosis (data not shown), therefore the clinical groups were combined for further analysis. When analyzed quantitatively and corrected for multiple comparisons, Rv1986 pool 1 and 2 stimulated significantly higher levels of IL2sR, TNF, IL-10, IL-13, MIP-1 alpha and MIP-1 beta than CFP-10, and similar levels of RANTES. Levels of IL-13 were very modest (Figure 4A, B and C). Thus the Rv1986 peptides were associated with a distinct pattern of cytokine production other than IL-2 when compared to CFP-10.

### T cell recognition of *M. tuberculosis* H37Rv with and without Rv1986

**Figure 5:**
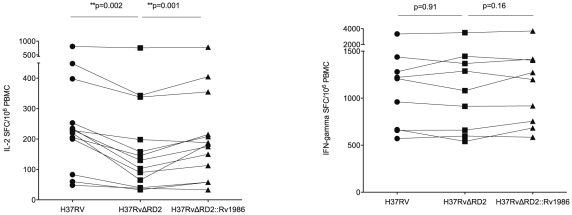
Live recognition of M. tuberculosis H37Rv, H37RvΔRD2 and H37RvΔRD2::Rv1986. PBMC from persons with latent tuberculosis were co-cultured with *M. tuberculosis* H37Rv (λ), H37RvΔRD2 (ν) and H37RvΔRD2::Rv1986 (σ) (complemented with Rv1986) for IL-2 and IFN-γ ELISpot. Results are represented as SFC/10^6^ PBMC. Panel A shows IL-2 SFC response. All 3 strains induced substantial IL-2 response (>30 SFC/10^6^ PBMC) in all donors. The median IL-2 SFC to MTB H37Rv was highest, followed by H37RvΔRD2*::*Rv1986 and the H37RvΔRD2 mutant. Panel B shows IFN-γ SFC response, which was much stronger than the corresponding IL-2 response. All 3 strains induced similar levels of IFN-γ response.

We next determined whether there was any difference in the IL-2 and IFN-γ responses to live strains of MTB in which Rv1986 was intact or deleted. 13 persons with latent tuberculosis were tested (only 9 patients for IFN-γ due to limitation in cell numbers). Although the overall IFN-γ SFC response to these MTB strains was much stronger than the IL-2 response, there was no significant difference in IFN-γ response between these strains (Figure 5B). The IL-2 SFC response to MTB H37Rv was significantly higher than to the H37RvΔRD-2 mutant (median 228 SFC/10^6^ PBMC, IQR 142–325 vs. 130 SFC/10^6^ PBMC, IQR 53–268; p = 0.002) and complementation by Rv1986 alone substantially restored the SFC response (183 SFC/10^6^ PBMC, IQR 86–285; p = 0.002, when compared to H37RvΔRD-*2*. Figure 5A).

### Response of HIV infected persons to Rv1986, ESAT-6 and CFP-10

**Figure 6:**
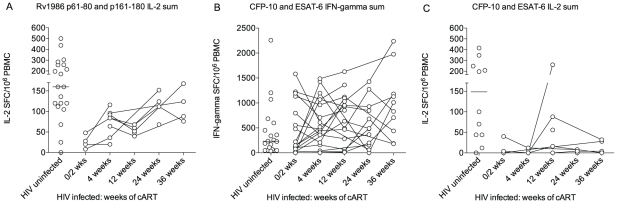
Response of HIV infected persons to Rv1986, ESAT-6 and CFP-10. The IL-2 to Rv1986 and IFN-γ response to CFP-10 and ESAT-6 before and during the course of antiretroviral therapy was compared. The respective responses were summed for analysis and compared to the values previously obtained for HIV uninfected persons of similar background, age and sex. (A) The IL-2 response to the peptides of Rv1986 was significantly lower in HIV infected persons prior to cART than in HIV uninfected persons. A significant increase in response occurred during cART therapy (p = 0.005 Kruskal-Wallis test). (B) The IFN-γ response to ESAT-6 and CFP-10 was not significantly lower in HIV infected persons prior to cART than in HIV uninfected persons The trend in median response during cART therapy was not significant (p = 0.22). (C) The IL-2 response to the peptides of ESAT-6 and CFP-10 was significantly lower in HIV infected persons prior to cART than in HIV uninfected persons (p = 0.02). No significant increase in response occurred during cART therapy.

The CD4 deficiency caused by HIV infection is the greatest recognized predisposing factor to tuberculosis and conversely antiretroviral therapy (cART) reduces susceptibility by suppressing viral replication and allowing CD4 recovery [29]. We reasoned it would therefore be of interest to compare the IL-2 to Rv1986 and IFN-γ and IL-2 response to CFP-10 and ESAT-6 before and during the course of antiretroviral therapy. As the IFN-γ response to Rv1986 had not been prominent in HIV-1 uninfected persons this was not assayed. The ELISpot response of 19 HIV infected persons without evidence of active tuberculosis was therefore tracked longitudinally over the first 36 weeks of therapy. All patients experienced CD4 increases and suppression of HIV replication during cART. We could not sample all time points and patients for both cytokines due to limitation in the number of cells. Figure 6 shows results of patients whose IL-2 and IFN-γ response to CFP-10 and ESAT-6 was assayed at least twice and 9 patients in whom the corresponding IL-2 response to peptides p61–80 and p161–180 could be determined. Peptide responses were summed for analysis and compared to the values obtained from 20 HIV uninfected persons of similar background, age and sex (i.e. those shown in Figure 2). The IL-2 response to the peptides of Rv1986 was significantly lower in HIV infected persons prior to cART (median 24, IQR 11–43) than in HIV uninfected persons (median 160, IQR 114–256, p = 0.009, Figure 6A). A significant increase in response occurred during cART therapy such that the median at 36 weeks increased to 106 (IQR 79–157, p = 0.005). By contrast the IFN-γ response to ESAT-6 and CFP-10 was not significantly lower in HIV infected persons prior to cART (median 147, IQR 50–965) than in HIV uninfected persons (232, IQR 56–563, p = 0.84). Whilst the median response did increase during cART therapy, the overall trend was not significant p = 0.22, Figure 6B). The IL-2 response to the peptides of ESAT-6 and CFP-10 was significantly lower in HIV infected persons prior to cART (median 2, IQR 0–31) than in HIV uninfected persons (median 148, IQR 44–323, p = 0.02, Figure 6C). No significant increase in response occurred during cART therapy. Taken together these findings indicate the decreased IL-2 to Rv1986 response prior to therapy correlates with increased susceptibility better than the IFN-γ response to CFP-10/ESAT-6; and that the partial but significant recovery of IL-2 to Rv1986, but unchanged IFN-γ response to CFP-10/ESAT-6 also correlates with the recognized decrease in tuberculosis susceptibility that is conferred by cART.

## Discussion

We have analyzed whole genome-based transcriptional profiles of *M. tuberculosis* subject to prolonged hypoxia to guide the discovery of potential antigens. Because the diagnostic potential of species-specific proteins is greatest we focused our initial consideration on two genes upregulated during hypoxia that are absent from all *M. bovis* strains including BCG by virtue of being RD11-encoded (Rv2658c and Rv2659c) [30], [31]. We also investigated the RD2-encoded Rv1986 because it is absent from most commonly used BCG strains. When compared to the well-characterized immunodominant and species-specific molecules ESAT-6 and CFP-10, RD11 proteins had inferior ability to restimulate IFN-γ from T cells of persons sensitized by either latent or active tuberculosis. However a striking finding was the immunodominance of Rv1986 for the IL-2 recall response, directed narrowly at two epitopic regions. The quantitative IL-2 response to Rv1986 was several times greater than the corresponding IFN-γ response to either ESAT-6 or CFP-10 (Figure 1C). Our findings suggest Rv1986 to be a major target of long lived CD4+ central memory T cells and that the Rv1986 peptides are associated with a distinct pattern of cytokine production when compared to CFP-10. There was significant recovery of IL-2 response to the peptides of Rv1986 than of IFN-γ response to ESAT-6 or CFP-10 during the course of cART in HIV infected persons. We also showed that deletion of Rv1986 from the genome of *M. tuberculosis* substantially decreases its ability to restimulate IL-2 secretion. These interesting findings are potentially important when considering vaccine-induced and natural immunity to tuberculosis and how immunodiagnosis may be improved.

One hypothesis we were interested to test is whether, by virtue of upregulation during hypoxia, proteins encoded by such genes would be preferentially recognized by latently infected persons. With the exception of the weak IFN-γ response to Rv2659c pool 2 (Figure 1A) this proved not to be the case. Hypoxia does characterize tuberculous granulomas *in vivo* [18] but it is increasingly re-appreciated that both active and latent tuberculous lesions exhibit a dynamic spectrum of overlapping morphologies [32], [33], [34], [35], [36], [37], [38] and that hypoxic lesions likely occur in both clinical circumstances. A link between transient increases in transcript abundance during hypoxia and the immunogenicity of *dos*R regulated proteins has also been attempted and the term ‘latency antigen’ has been introduced [20]. A dominant antigenic target that is *dos*R regulated is Acr1 encoded by Rv2031c and under some assay conditions we, and subsequently others, have documented preferential T cell recognition of Acr1 by latently infected people [19], [21]. Preferential recognition of Acr1 by latently infected persons was not observed in this study (Figure 1) nor in our previous IFN-γ ELISpot analysis [22], which is in fact consistent with expression of Acr1 throughout experimental infection [39], [40]. Conversely a quantitatively higher IFN-γ response to the RD1 encoded CFP-10 and ESAT-6 antigens has sometimes been associated with active disease [41], [42], [43], [44], attributed to the secretion of these proteins by actively replicating bacilli. We did not however observe a higher response in active tuberculosis compared to latently infected persons. Differences in infection pressures between low and high incidence areas might feasibly contribute to these differences: the clinical environment in which we conducted this study suffers an extraordinarily high tuberculosis incidence of ∼1500/100,000 with much ongoing transmission [45]. It is also interesting to note that whilst the transcriptomic data showed a fold decrease in ESAT-6 and CFP-10 during hypoxia, the absolute abundance of these transcripts remained high (Table 1). Expression of ESAT-6 and CFP-10 under a variety of conditions is in agreement with other *in vitro* expression data [14], [46] and adds to data suggesting these molecules may play a role in bacillary persistence as well as active infection [4]. The availability of expression profiles from latently infected human tissue rather than from axenic *in vitro* culture might provide a better starting point for antigen discovery.

Although IFN-γ is essential to human defense against mycobacteria, it is increasingly recognized that assay of PBMC secretion of IFN-g is a poor correlate of protection in field studies of tuberculosis [47]. Greater attention to markers, such as IL-2, that might better reflect immunological memory is now being paid and formed the basis for our investigation by ELISpot assay of this cytokine [28], [41], [48], although IL-2 secretion itself is not established as a better correlate of protection than IFN-γ. Polyfunctional T cells that secret multiple cytokines are considered a potential correlate of protection in tuberculosis [49], [50] although the finding that such cells are expanded in tuberculosis patients rather than healthy contacts has been interpreted by some to indicate a role in pathology rather than protection [51]. In this context our finding that Rv1986 was so dominant for the single positive IL-2 response yet elicited modest IFN-γ secretion was striking. The cytokine phenotype of antigen specific T cells is greatly influenced by co-stimulation and the cytokine milieu [52], [53]. However it has also been suggested that the overall affinity of the TcR-peptide-MHC interaction may play a role as well [54], [55]. Interestingly an epitope in Rv1986 with an anchor at position 167 (corresponding with p161–180) is predicted for several DRB1*03, *04, *08, *11 and *13 alleles [56]: a promiscuous binding ability that has been noted for other *M. tuberculosis* epitopes [55] and which might contribute to the almost universal response we observed to this peptide. Rv1986 is a putative membrane protein that is recognized by T cells from *M. bovis* infected cows [57]. Although the responses to other RD-2 encoded antigens has been previously evaluated in humans [58], [59], [60], [61], the human T cell response to Rv1986 has not been reported. Our finding that Rv1986 is a dominant target of IL-2 secreting memory T cells suggests that this recall response could contribute to protective immunity.

Our findings also bring a novel twist to an old story: the partial and globally variable efficacy of BCG vaccine [62], [63]. Henao-Tamayo and colleagues recently investigated the vaccine efficacy of BCG Pasteur concluding its ability to induce central memory T cells in the lung was poor perhaps contributing to its partial efficacy [64]. Although another recent study noted no experimental difference in short-term protective efficacy in Guinea Pigs between RD2- negative (e.g. BCG Pasteur) and RD2-positive (e.g. BCG Japan) strains [65] our discovery that a major target of the human IL-2 response is absent from the most commonly used strains is intriguing. Whilst the *in vitro* diagnostic potential of the two dominant peptides we have uncovered is considerable, the most important consequence of this work may be to re-evaluate by clinical trials whether BCG strains with and without RD2 vary in clinical efficacy.

## Methods

### Microbial culture conditions, hypoxia model and microbial RNA analysis

These techniques have been extensively described before [25]. Briefly, exponential phase cultures grown in rolling culture to an OD600 of 0.3 were diluted to a starting OD of 0.1 with warm media. This starting culture was transferred to a constantly stirred 1 liter flask, 500 mL of this starting culture per flask. Hypoxia was generated by introducing a constant flow of nitrogen with trace amounts of oxygen (0.2% O2), leading to bacteriostasis. Samples were taken before hypoxia, at four hours, and after 1, 4, and 7 days of exposure to hypoxia. RNA was isolated from these samples using bead beating in the presence of Trizol, followed by chloroform extraction and precipitation of RNA. The RNA was further cleaned using an RNeasy kit purchased from Qiagen. Approximately 3 µg of purified RNA was converted to cDNA using Superscript III (Invitrogen). Aminoallyl dUTP was included in the cDNA reaction, and subsequently conjugated to reactive Cy dye esters. The aerobically growing transcriptional profiles were directly compared to each subsequent hypoxic time point by cohybridization on the same microarray slide. The microarray slides and protocols were provided by the Pathogen Functional Resource Center at the J. C. Ventner Institute as part of their NIAID contract N01-AI-15447. Slides were scanned with a GenePix 4000B purchased from Axon Technologies. Raw background subtracted intensities were normalized to SigA to provide an approximate measure of transcript abundance.

### Human subjects

The University of Cape Town research ethics committee approved this study (REC 296/2007). Written informed consent was provided by study participants. Patients with active or latent tuberculosis were recruited at the Ubuntu clinic at Khayelitsha site B, South Africa. All were of Xhosa ethnicity. Active tuberculosis (ATB) was defined by smear positivity for and/or culture of *M. tuberculosis* from one or more sputum specimens. Latent tuberculosis (LTBI) was defined by transverse TST reactivity of >15 mm in response to 2 TU PPD (RT23) at 48–72 hours or an interferon-γ Enzyme linked immunospot (ELISpot) response to ESAT-6 or CFP-10 of >20 spot forming cells (SFC)/10^6^ PBMC in the absence of clinical symptoms or radiographic abnormality and with a negative sputum smear and culture for *M. tuberculosis*. All subjects underwent voluntary counseling and testing for HIV-1 infection and positivity was an exclusion criterion. ATB patients were sampled prior to commencing antitubercular chemotherapy. Known immunosuppression for other reasons, age <18 years and pregnancy formed other exclusion criteria. Another group of HIV-1 infected adults who were starting antiretroviral therapy, followed up for 36 weeks were also included as previously described in detail [29]. Patients with ATB and/or HIV infection were treated according to South African national guidelines. The baseline characteristics of subjects enrolled to the study are shown in Table 2.

**Table 2:** Characteristics of study participants.

	Active tuberculosis	Latent tuberculosis	Significance, p	HIV Infected	
**Number**	20	48		**Number**	19
**Median age**	32.5	20.7	<0.0001	**Median age**	35
**Sex (M:F)**	11M:9F	12M:36F		**Sex (M:F)**	10M:9F
**Sputum smear positive**	19 Positive1 unknown	NA	NA	**CD4 at recruitment (median)**	90
**Sputum culture positive**	18 Positive2 not available	NA	NA	**CD4 after 6 months of cART (median)**	209
**BCG vaccinated**	5 vaccinated6 Not vaccinated9 Don't know/data unavailable	23 vaccinated20 Not vaccinated5 Don't know/data unavailable		**Viral Load at recruitment (median)**	130,000 copies/ml
**Median Mantoux (IQR)**	NA	11 mm (IQR 8–20)	NA	**Viral Load after 6 months of cART (median)**	158 copies/ml

### Cell culture and ELISpot assays

Peripheral blood mononuclear cells (PBMC) were separated over Ficoll. Cells were frozen and stored in liquid nitrogen until analyzed in batches. A total of 2.5×10^5^ PBMC were added in 100 µl of RPMI/10%FCS (R10)/well for ELISpot and in 200 µl of R10/well for cell culture. Antigenic stimuli were in the form of pools (maximum 13 peptides in a pool) of 20-mer peptides overlapping by 10 residues with each peptide used at a final concentration of 10 µg/ml. Peptides were purchased from Peptide Protein Research Ltd, Oxford, UK and from Pepscan Presto B.V, Netherlands. Peptides were HPLC purified and their mass verified by Mass spectrometry. Control stimuli for ELISpot included anti-CD3 mAb CD3-2 at 100 ng/ml final concentration and unstimulated wells.

The interferon-γ ELISpot assay was performed as previously described with slight modifications [29]. Ninety-six well precoated ELISpot plates, mAb 1-D1K (Pre-coated One-step, Mabtech; 3420-2ATP-10) were washed with sterile PBS, blocked with R10 for ≥30 min at room temperature. The blocking medium was removed and the PBMC were set up with respective antigenic stimuli. After incubation for16 h at 37°C with 5% CO2, plates were washed with PBS, and 100 µl of secondary antibody, mAb 7-B6-1-ALP conjugate at 0.5 µg/ml final concentration in PBS containing 0.5% FCS was added. After 2 h of incubation at room temperature, 100 µl of filtered ready to use substrate solution (BCIP/NBT-plus) was added and developed until spots emerged, washed with tap water and allowed to dry.

For the IL-2 ELISpot, 96- well polyvinylidene difluoride membrane based plates, type ELIIP (MAIPSWU10; Milipore Corp), were activated by a brief treatment with 70% ethanol, coated overnight at 4°C with 15 µg/ml of mAb IL2-I (Mabtech; 3440-2AW-Plus), and blocked with R10 for ≥30 min. The blocking medium was removed and the PBMC were set up with respective antigenic stimuli. After 16-h incubation at 37°C in 5% CO2, the plates were washed, 100 µl of detection antibody (IL-2-II-biotin) at 1 µg/ml in PBS containing 0.5% FCS added and incubated at room temperature. After 2 hrs, 100 µl of Streptavidin-ALP 1∶1000 in PBS-0.5%FCS was added and incubated at room temperature. After 1 h, 100 µl of substrate solution (BCIP/NBT-plus) was added and developed until distinct spots emerged. Plates were washed with tap water and allowed to dry. Spot forming cells were enumerated by immunospot counter (CTL, Cellular Technology Ltd) and confirmed by microscope (X4). Results are quoted as cytokine spot forming cells (SFC)/10^6^ PBMC.

The ELISpot (IFN-γ and IL-2) experiments using live *M. tuberculosis* strains H37Rv, H37RvΔRD2 and H37RvΔRD2::Rv1986 (complemented by Rv1986), were performed as described above and previously [66] with 200,000 PBMC/well cultured for 16–18 hrs in the presence of 200,000 live bacteria/well (in duplicate wells).

### Recombinant MTB strains

The MTB H37RvΔRD2 strain (RD-2 mutant) was prepared using homologous recombination and sucrose counter-selection as previously described [15]. This mutant was then electroporated with either the empty plasmid pMV306 or the same plasmid into which Rv1986 from H37Rv had been cloned. This gene was expressed under its native promoter. This resulted in the MTB H37RvΔRD2::pMV control (H37RvΔRD2) and MTB H37RvΔRD2::Rv1986 (complemented with Rv1986) strains, which were grown in 7H9 + ADC + 0.05% Tween 80 + Kanamycin (25 ug/ml) and preserved as 25% glycerol stocks.

### Intracellular cytokine staining assay (FACS)

1.5–2×10^6^ PBMC were incubated with the two Rv1986 peptides (residues 61–80 and 161–180) at 10 µg/ml each (i.e. 20 µg/ml peptide in total) or a pool of 21 peptides from CFP-10 and ESAT-6 at 2 µg/ml each (i.e. 42 µg/ml peptides in total) at 37°C. Control stimuli included SEB as positive control at10 µg/ml and unstimulated cells as negative control. After 2 hrs, Brefeldin A at 5 µg/ml (Sigma, St. Louis, MO) was added to capture the newly formed cytokines in the Golgi apparatus. After 16 h incubation (in total), the cells were washed with PBS (1X). For 8 color surface and intracellular staining the cells were first permeabilized, and fixed using Cytoperm/cytofix buffer (BD) for 20 min at 4°C, washed with BD Perm/wash and stained with antibody cocktail in BD perm/wash for 1 hr at 4°C. The antibodies used were as follows: CD3-Pacific Blue (1 µl/tube), CD4 QDot605 (0.5 µl/tube), CD8 Cy5.5PerCp (3 µl/tube), IFN-γ Alexa700 (1 µl/tube), IL-2 FITC (5 µl/tube), TNF Cy7PE (5 µl/tube), CD45RA- APC (3 µl/tube), CD27-PE (3 µl/tube), all of which were purchased from BD BioSciences. 10^6^ cells were acquired on LSR II flow cytometer (BD Bioscience). Cell doublets were excluded using forward scatter area vs. forward scatter height parameters. Unstained cells and single-stained mouse calibration beads were used to calculate compensations for every run. Data analysis was performed using FlowJo v 8.8.2 (Tree Star), Pestle v 1.6.1 (NIH) and Spice v 5.05013 (NIH). We defined T cell phenotypes based on the surface markers CD45RA and CD27: Central memory cells (TCM) as positive for CD27 and negative for CD45RA; effector memory (TEM) are negative for both CD27 and CD45RA and Terminally differentiated T cells (Tdiff) are negative for CD27 and positive for CD45RA. The results are expressed as the percentage of CD3+ CD4+ T cells.

### Multiplex cytokine analysis

Bioplex, mixed-to-order panel (premixed multiplex panel) from Biorad was used for multiplex cytokine analysis. The assay was carried out according to the manufacturer's instructions. Briefly, the 96- well filter plate was pre- wet with 150 µl of Biorad assay buffer and the buffer removed by vacuum filtration. 50 µl of multiplex bead working solution was added to the wells and the buffer removed. 100 µl of Bioplex wash buffer was added to each well and washed twice and the buffer removed. 50 µl of standard and sample was added to the respective wells, the plate was sealed and then covered by aluminum foil and placed over a microplate shaker. The speed of the shaker was increased to 1100 RPM for 30 sec and then reduced to 300 RPM for 30 min, incubation at room temperature. After incubation, the plates were washed 3 times with Bioplex wash buffer. 25 µl of Bioplex detection antibody working solution was added, and incubated for 30 min as above on the microplate shaker at room temperature. The plates were washed 3 times with Bioplex wash buffer and 50 µl of streptavidin-PE was added, and incubated for 10 min, washed 3 times with Bioplex wash buffer. Beads were resuspended with 125 µl of Bioplex assay buffer, mixed over the microplate shaker at room temperature at 1100 rpm for 30 sec and read on the Bioplex suspension array system.

### Statistical analysis

The normality of data was assessed by the D'Agostino and Pearson omnibus test using Graphpad Prism 5.0 software (www.graphpad.com). Parametric continuous variables were assessed by student's paired and unpaired t-tests, and non-parametric by Wilcoxon matched pairs, Kruskal Wallis test with Dunn's post test correction or Mann Whitney U tests. Contingency analysis was by Fisher's exact test of probability.
